# The Global Parkinson's Genetics Program (GP2): Advancing genetic discovery and capacity building worldwide

**DOI:** 10.1177/1877718X261452868

**Published:** 2026-05-20

**Authors:** Victor Flores-Ocampo, Alexandra Zirra, Yi Wen Tay, Vesna van Midden, Ignacio F Mata, Alastair J Noyce, Maria Teresa Periñan, the Global Parkinson's Genetics Program (GP2)

**Affiliations:** 1 School of Biomedical Sciences, Faculty of Medicine, 420004 The University of Queensland, Brisbane, QLD, Australia; 2 Brain & Mental Health Program, 56362 QIMR Berghofer Medical Research Institute, Brisbane, QLD, Australia; 3 Centre for Preventive Neurology, 105714 Wolfson Institute of Population Health, 4617 Queen Mary University of London, London, UK; 4 Department of Biomedical Science, Faculty of Medicine, 595410 University of Malaya, Kuala Lumpur, Malaysia; 5 Department of Neurology, 398172 University Medical Centre Ljubljana, Ljubljana, Slovenia; 6 Genome Sciences and Systems Biology, 196246 Cleveland Clinic Research, Cleveland Clinic, Cleveland, OH, USA; 7 Unidad de Trastornos del Movimiento, Servicio de Neurología y Neurofisiología Clínica, Instituto de Biomedicina de Sevilla, Hospital Universitario Virgen del Rocío/Consejo Superior de Investigaciones Científicas (CSIC)/Universidad de Sevilla, Seville, Spain

**Keywords:** Parkinson's disease, genetics, capacity building, research training, underrepresented populations

## Abstract

The Global Parkinson's Genetics Program (GP2) is an international initiative funded by Aligning Science Across Parkinson's (ASAP), in partnership with the Michael J Fox Foundation for Parkinson's Research (MJFF), to accelerate genetic discovery and improve ancestral representation in Parkinson's disease (PD) and related diseases through collaboration, open data sharing, and research capacity building. Since its launch in 2020, GP2 has assembled the largest and most ancestrally diverse PD dataset to date, integrating genotyping, sequencing, and harmonized clinical data from over 240 cohorts worldwide. Through its structured monogenic and complex disease networks, the program spans rare and common variant discovery to advance understanding of PD genetics. Recent GP2-supported studies have identified more than 50 novel genetic risk factors for PD, including a remarkably common *GBA1* risk variant among people of African ancestry, and have confirmed new candidate causal genes such as *RAB32*. Ongoing efforts include whole-genome burden testing, multiple ancestrally-diverse genome-wide association studies (GWAS), polygenic risk modeling, and expansion into atypical Parkinsonism and prodromal cohorts. Beyond discovery, GP2 has invested extensively in research infrastructure and training, supporting more than 270 early-career investigators through workshops, hackathons, and a trainee-to-trainer mentorship framework. These initiatives build local capacity and empower researchers, particularly in underrepresented regions, to lead future genetic studies. GP2 provides an equitable, collaborative model for accelerating the field's understanding of the genetics of PD and related disorders. Continued expansion will enhance population diversity, refine mechanistic insights, better delineate disease onset and progression, and advance progress toward precision medicine across the Parkinsonian spectrum.

**Plain language summary title:**

GP2: Genetics and Capacity Building Worldwide

## Plain language summary

Why was this research done?

Parkinson's disease (PD) is a common brain condition that affects movement and quality of life. While genes are known to play a role in PD, most genetic studies have focused on people of European ancestry. This means we know less about how PD affects other populations and how genes influence risk in different parts of the world.

What is the Global Parkinson's Genetics Program (GP2)?

Launched in 2020, GP2 is a global research effort supported by the Aligning Science Across Parkinson's (ASAP) initiative and the Michael J. Fox Foundation. Its goal is to better understand the genetic causes of PD by collecting data from over 250,000 people worldwide, with a focus on including underrepresented populations.

What did the researchers do?

GP2 brings together more than 400 research groups from 80+ locations. It has already collected genetic and clinical data from over 100,000 individuals. The program studies both common and rare genetic variations linked to PD and related conditions like dementia with Lewy bodies and multiple system atrophy. GP2 also builds research tools, provides open access to data, and supports training for scientists, especially those in low-resource settings.

What did they find?

So far, GP2 has helped identify over 50 new genetic risk factors for PD. Some of these are especially relevant to people of African, Latin American, or South Asian ancestry. GP2 has also launched studies on early-stage PD, genetic forms of the disease, and the progression of symptoms.

Why does this matter?

GP2 is helping to create a more complete, global picture of PD. Its work supports fairer, more effective research and lays the groundwork for treatments that benefit all people, regardless of where they live or their ancestry.

## Overview of GP2

The Global Parkinson's Genetics Program (GP2, gp2.org) was launched in January 2020 as an ambitious five-year initiative funded by Aligning Science Across Parkinson's (ASAP, https://parkinsonsroadmap.org/) and implemented by The Michael J. Fox Foundation for Parkinson's Research. The project was created to address the need for a greater understanding of genetic risk factors in Parkinson's disease (PD) and related disorders (e.g., Corticobasal syndrome, Progressive Supranuclear Palsy, Dementia with Lewy Bodies, and Multiple System Atrophy) across diverse ancestries, as previous research had largely focused on Western populations (European ancestry), neglecting global genetic diversity. The ambition at the outset was to generate genetic and associated clinical data in over 150,000 participants worldwide, with a particular focus on underrepresented populations (including African, Asian, Middle Eastern, and Latin American populations). The central mission of GP2 is to dramatically expand our understanding of the genetic basis of PD and related disorders in a global context. To achieve this, we built a global research community with the tools and infrastructure to address emerging research needs and questions.^1,2^

GP2 is built on four core pillars: global collaboration and capacity building; open and equitable data access; transformative, cutting-edge genetic methods and analyses; and training the next generation of researchers and leaders. These pillars ensure rigorous, sustainable, and inclusive progress in PD neurogenetics. By developing cloud-based data platforms, harmonized analysis pipelines, and regional training hubs, GP2 empowers researchers worldwide, particularly in low-resource settings, to contribute directly to global discoveries and leadership in the field.

GP2 is uniquely positioned to expand our understanding of the genetic landscape of PD and accelerate therapeutic discovery, not only through data collection and harmonization, but also by strengthening international collaboration and building a cooperative and highly motivated research community capable of addressing large knowledge gaps. This is particularly relevant since PD genetics encompasses both rare, high-impact variants in single genes that cause PD and common variants across multiple genes that modulate disease risk. Unraveling these aspects requires different but complementary approaches.

Monogenic PD results from pathogenic variants in single genes, typically *SNCA*, *LRRK2*, *VPS35*, *RAB32*, *PRKN*, *PINK1*, and *DJ-1*, which are sufficient to cause disease in a Mendelian fashion, often in familial or early-onset cases.^3^ Studies of these genes have elucidated central pathogenic processes, implicating mitochondrial quality control, lysosomal–autophagy pathways, and protein aggregation biology.^4–7^ In contrast, polygenic PD reflects the aggregate contribution of numerous common variants of small effect sizes distributed across the genome. Large-scale genome-wide association studies (GWAS) have now identified more than 150 risk loci, underscoring the importance of genetic background and its interaction with the environment in modulating susceptibility.^8,9^ Recognizing that these two faces of genetic risk (rare, high-penetrance variants and common, low-effect variants) exist more on a spectrum than on opposite sides of a coin motivates complementary discovery strategies and analytical frameworks.

GP2 is organized into working groups, overseen by an Operations Committee, and structured around two core networks that mirror the spectrum of genetic risk: the Monogenic Network and the Complex Disease Network. The Monogenic Network focuses on identifying rare, high-impact genetic causes of PD and includes two working groups (WGs): a Data Generation WG responsible for the collection and analysis of familial and early-onset samples, and a Data Analysis WG tasked with discovering novel causative variants and genes.^10,11^ The Complex Disease Network interrogates polygenic contributors by integrating clinical and genotyping data across diverse ancestral populations and comprises the Cohort Integration WG, responsible for harmonizing and unifying cohort-level datasets, and the Data Analysis WG, which performs genome-wide association and polygenic risk analyses. As part of the program's ongoing expansion, the Prodromal WG spans both networks, integrating data from cohorts at the earliest stages of disease to improve understanding of the genetic influences on PD onset and progression.

Complementing both networks are two additional cross-cutting groups: the Underrepresented Populations (URP) WG and the Training and Networking WG. The URP WG works to enhance representation of non-European ancestries in PD research by supporting recruitment and data generation in these populations. The Training and Networking WG focuses on building a global community of early-career researchers and clinicians through regional training initiatives and the development of free online learning materials available on the GP2 Learning Platform (https://training.gp2.org/). Together, these groups address a core component of the GP2 mission: democratizing access to knowledge and fostering a diverse, internationally engaged research community.

To ensure efficient operations and adherence to open-science principles, GP2 also includes a suite of WGs: the Operations and Compliance WG, which oversees data and biosample sharing; the Data and Code Dissemination WG, which reviews and releases analysis pipelines to promote reproducibility; the Project Proposal, Approval, and Execution WG, which evaluates and facilitates project proposals; and the Publication Development WG, which coordinates manuscripts using GP2 data and maintains scientific quality through an internal peer-review process.

Since its formation, GP2 has grown into a large-scale, open-science initiative that brings together hundreds of organizations and around 400 research cohorts worldwide to create an inclusive and collaborative research community for PD. As of December 2025, GP2 includes 436 research cohorts across 84 unique locations, with 103,786 samples already analysed and more than 343,123 expected at completion, more than doubling the original goal of 150,000. The initiative also involves 59 centres actively contributing to monogenic research, ensuring representation across both rare and complex genetic forms of PD. In parallel with its scientific objectives, GP2 has established a strong educational and capacity-building framework, engaging approximately 410 trainees globally, of whom 225 are from URP countries. Collectively, these achievements underscore the scale and impact of GP2's outreach and its ongoing commitment to fostering an equitable, collaborative, and sustainable global PD genetics community.

## Progress and key findings

**Figure 1.**
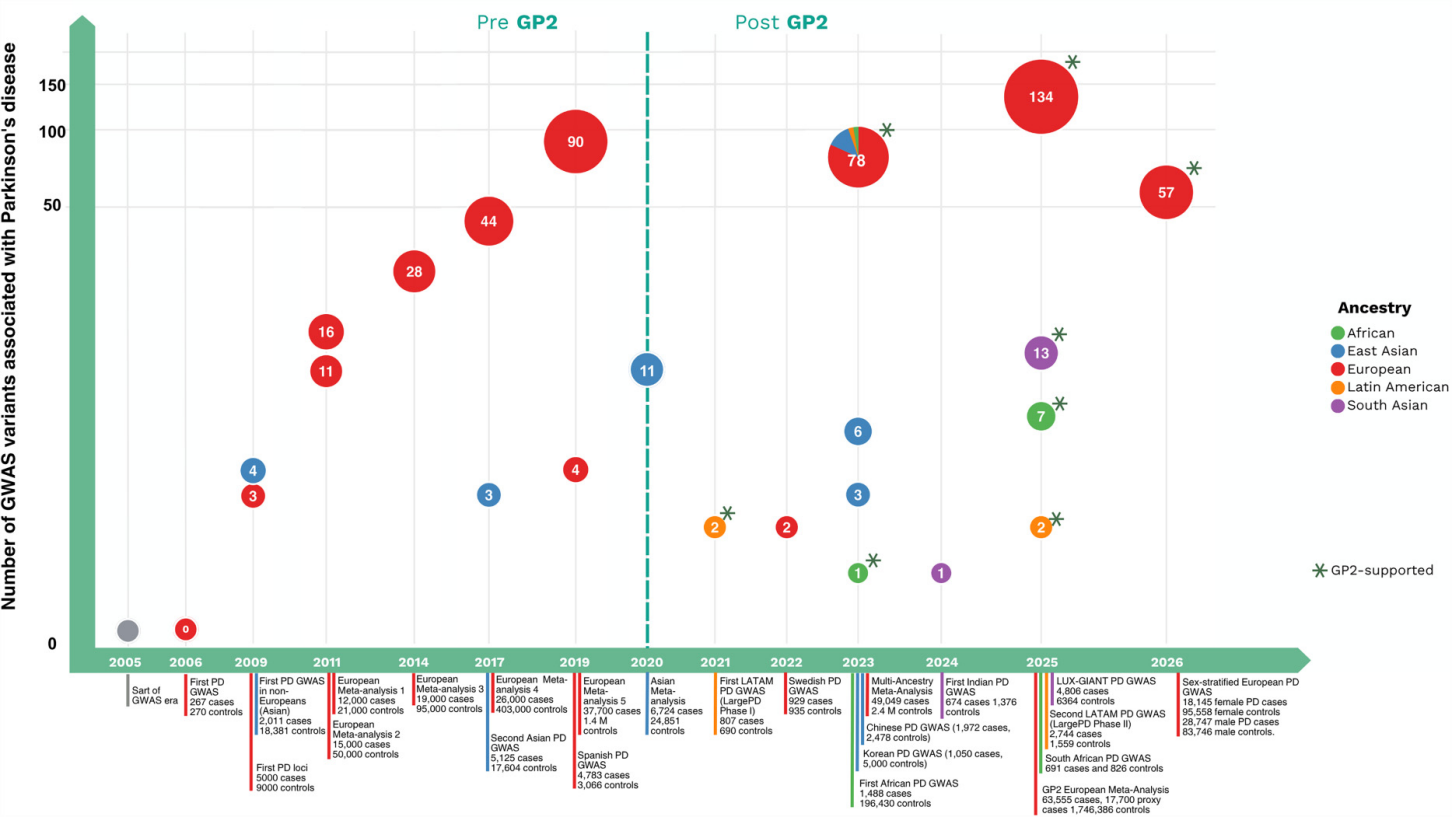
Parkinson’s disease GWAS discoveries over time. The figure depicts the number of genome-wide significant variants identified in Parkinson’s disease (PD) genome-wide association studies (GWAS) and meta-analyses published between 2006 and 2026. Each bubble represents a GWAS or meta-analysis, with the y-axis indicating the number of reported PD-associated variants. Bubble size scales proportionally with the number of variants identified. Colors denote the ancestry of the study population: European (red), East Asian (blue), African (green), Latin American (LATAM) (orange), and South Asian (purple). Studies supported by the Global Parkinson’s Genetics Program (GP2) are indicated with a green asterisk (*). The data highlight the increasing scale and ancestral diversity of PD genetic research over time.

GP2 has defined a set of core analyses designed to accelerate discovery and address key gaps in our understanding of PD genetics. These efforts leverage GP2's diverse cohorts to examine monogenic variants alongside common and rare polygenic determinants of disease risk, age at onset (AAO), progression, and ancestry-specific susceptibility. Together, these analyses encompass the largest European PD GWAS to date, the first coordinated multi-ancestry analysis, and an expanding set of ancestry-specific studies^8,12–14^ (Figure 1; Table 1).

**Table 1.** Chronological overview of key findings from GP2-supported studies.

Study (year)	Population	Key findings	DOI
First Latin American PD GWAS (LARGE-PD Phase 1) (2021)	807 PD cases; 690 controls	Identified a genome-wide significant association at the *SNCA* locus	10.1002/ana.26153
First PD GWAS in African and African admixed populations (2023)	1488 PD cases; 196,430 controls	Identified an ancestry-specific PD risk variant in *GBA1* (rs3115534-G)	10.1016/S1474-4422(23)00283-1
Multi-ancestry PD GWAS (MAMA) (2023)	49,049 PD cases; 18,618 proxy cases; 2,458,063 controls (multi-ancestry)	Identified shared and ancestry-specific PD loci, including 12 novel associations	10.1038/s41588-023-01584-8
Functional study of *GBA1* rs3115534-G variant (2024)	African ancestry individuals	Showed that rs3115534-G disrupts a branchpoint motif affecting splicing	10.1038/s41594-024-01423-2
*RAB32* Ser71Arg in autosomal dominant PD (2024)	Familial PD cases	Implicated *RAB32* Ser71Arg as a novel PD-associated variant	10.1016/S1474-4422(24)00121-2
Largest European PD GWAS (2025)	63,555 PD cases; 17,700 proxy cases; 1,746,386 controls	Identified 134 independent loci, including 59 novel associations	10.1101/2025.03.14.24319455
First PD GWAS in India (2025)	4806 PD cases; 6364 controls	Identified 13 genome-wide significant loci, including two novel associations (*ATP10A* and *MICD)*	10.1101/2025.02.17.25322132
Global Landscape of Genetic Variation in PD (2025)	58,559 PD cases; 5278 other NDD; 512 relatives; 41,239 controls (multi-ancestry)	Revealed ancestry-specific differences in the frequency and spectrum of pathogenic variants	10.1101/2025.07.08.25330815
Second Latin American PD GWAS (2025)	Phase 1 + 4401 additional samples	Identified a likely novel PD risk locus in *ITPKB*, common in American admixed but rare in Europeans	10.1101/2025.07.18.25331793
South African PD GWAS (2025)	691 PD cases; 826 controls	Identified a novel locus in *AKAP6* (rs17098735-T)	10.1101/2025.08.01.25331910

An early example is the multi-ancestry GWAS meta-analysis of PD, comprising 49,049 PD cases, 18,785 proxy cases, and 2,458,063 controls across European, East Asian, Latin American, and African ancestries. This study identified 78 genome-wide significant loci (including 12 novel loci) and nominated 25 putative risk genes. These results underscore both shared and population-specific features of the PD genetic landscape. While many loci show consistent effects across populations, differences in allele frequencies, effect sizes, and fine-mapping resolution reflect ancestry-specific genetic architectures. The authors further emphasized that much remains to be discovered about the determinants of PD pathogenesis in non-European populations.^8^ A complementary analysis using population-attributable risks highlighted how common genetic variation differentially contributes to PD and Alzheimer's disease across ancestries.^15^

In addition to initial multi-ancestry analyses, which will rapidly expand now that genotyping data has been produced at scale, GP2 has supported a series of regional GWAS efforts that provide critical insight into ancestry-specific genetic architecture. In 2021, the LARGE-PD consortium (established prior to GP2) reported the first GWAS of PD in Latin America, analysing 1497 participants and identifying a genome-wide significant association at the *SNCA* locus (Phase 1).^16^ Recruitment in Latin America has since more than doubled, and a follow-up GWAS incorporated the Phase 1 dataset with an additional 4401 samples. This expanded study applied multiple analytical approaches to account for genetic relatedness, global ancestry, and admixture. These efforts addressed the complex admixed architecture of Latin American populations and identified a plausible novel PD locus in *ITPKB,* nearly absent in Europeans but common among Peruvians and other admixed groups.^12^

In South Asia, the Lux-GIANT (Luxembourg-German-Indian Alliance on Neurodegenerative Diseases and Therapeutics) consortium conducted the first PD GWAS in the region, encompassing 4806 PD cases and 6364 controls from India. The study identified 13 genome-wide significant loci, including two novel loci, and found evidence of a higher genetic burden of PD in the Indian population compared with Europeans. A meta-analysis with the multi-ancestry PD GWAS revealed three additional loci (*RLP8L1*, *TEC,* and *DSCAM).*^13^

GP2-supported activities in Africa have already yielded significant insights and represent a landmark effort to address the long-standing underrepresentation of African and African-admixed populations in PD genetics. This work is being advanced through major collaborative initiatives that aim to expand cohort recruitment and build the infrastructure needed for genetic research across Africa.^14^ The first GP2-supported GWAS in the region analysed 1488 PD cases and 196,430 controls from West Africa, revealing a novel risk locus at *GBA1* (rs3115534-G).^17^ Of note, rs3115534-G is much more common in individuals of African or African-admixed ancestry than in other populations (allele frequency = 0.16 in gnomAD and 0.21 in the African 1000 Genomes panel). Functional analyses suggest that rs3115534-G acts through a splicing-related mechanism rather than altering the protein sequence; it disrupts a branchpoint motif important for normal splicing, leading to exon skipping, reduced *GBA1* expression, and subsequent glucocerebroside activity.^18^ A subsequent GWAS in South Africans, using local ancestry models in 691 PD cases and 826 controls, identified a novel genome-wide significant locus in *AKAP6* (rs17098735-T). By modelling ancestry-specific effects, the study further identified three signals across ancestral components, including one near *WNT5A*/*LRTM1* and two implicating *CSMD1.*^19^

GWAS in Europeans continue to play a key role in refining the genetic architecture of PD. Most recently, the largest European PD GWAS meta-analysis to date was conducted by GP2, incorporating data from 63,555 clinically confirmed PD cases, 17,700 proxy cases, and 1,746,386 controls. The analysis identified 134 independent risk loci, including 59 novel associations, and performed subgroup analyses in Ashkenazi Jewish, Finnish, and Icelandic populations, as well as the broader European cohort. Functional annotation showed that PD-associated loci were enriched in brain tissues, particularly substantia nigra neurons and astrocytes, underscoring the value of large-scale analyses for refining population-specific risk profiles.^20^

GP2 has also undertaken complementary core analyses exploring additional aspects of disease biology. A major focus is AAO, assessing whether established risk variants influence the timing of disease manifestation. With support from GP2, the Parkinson's Families Project provides extensively phenotyped early-onset and familial PD cases for AAO analyses, with over 500 genomes sequenced through the GP2 Monogenic Network.^21^ Other research streams examine determinants of motor progression using quantitative measures such as MDS-UPDRS scores, Hoehn & Yahr staging, dementia incidence, and mortality. Collectively, these studies complement large-scale discovery efforts by addressing disease heterogeneity and linking genetic risk to clinically meaningful outcomes.

In parallel with advancing research on complex PD genetics, GP2 has prioritized monogenic PD as a foundational objective. This is exemplified by a GP2-supported study characterizing the global landscape of pathogenic and likely pathogenic as well as high-risk variants in established PD genes across 58,559 PD cases, 5278 participants with other neurodegenerative or movement disorder phenotypes, 512 unaffected family members of those individuals, and 41,239 controls from 11 ancestries, about 30% of whom were of non-European ancestry. The study revealed substantial ancestry-specific differences in variant frequencies and spectra, underscoring the importance of global representation in PD genetics research.^22^

An independent study leveraging GP2 data investigated *RAB32* Ser71Arg, a rare variant linked to autosomal dominant PD, using linkage, association, and functional analyses. The work confirmed its role as a disease-associated variant, demonstrated segregation within families, and showed that it disrupts *RAB32* function in experimental models, providing strong evidence that RAB GTPase dysregulation contributes to PD pathogenesis. The study also positioned *RAB32* within the broader context of *LRRK2* signaling.^23^

GP2 continues to integrate rare- and common-variant analyses through whole-genome burden testing, gene prioritization, and rare-variant aggregation approaches, including emerging efforts such as X-chromosome–wide analyses and evaluation of *PINK1*/*PRKN* heterozygous carriers, and studies of *LRRK2* and *GBA1* variant carriers to identify modifiers of disease risk and onset.

## Expansion plans and five-year outlook

GP2 was initially established as a 5-year program. However, based on early progress and emerging opportunities, its duration was extended from 2025 to 2029 to achieve several key objectives focused on expanding: 1) data collection in URP, 2) opportunities for WGS, and 3) collections in atypical Parkinsonian disorders and prodromal PD.

### Atypical Parkinsonism and prodromal disease

PD is just one cause of Parkinsonism, and misdiagnosis occurs in ∼15–20% of clinically diagnosed cases.^24,25^ Given the overlap in phenotype, management, and potential genetic determinants, it became evident that GP2 needed to expand beyond recruiting only PD cases and healthy controls. Atypical Parkinsonism (or Parkinson's plus disorders) includes multiple system atrophy (MSA), progressive supranuclear palsy (PSP), dementia with Lewy bodies (DLB), and corticobasal syndrome (CBS). These disorders are generally more aggressive, are associated with shorter survival, offer fewer symptomatic treatment options, and result in reduced quality of life for patients and caregivers.^26–31^ Classically, these diseases have been defined by their neuropathology, with PSP and corticobasal degeneration (CBD) being classified as tauopathies; MSA as a synucleinopathy; and DLB and PD as Lewy body disorders (also synucleinopathies). However, significant copathology is seen in both atypical Parkinsonism and idiopathic PD. Not only are Lewy bodies present in 10–20% of PSP and CBD, but tau-repeat deposition is also observed in 43–56% of MSA and DLB/PD cases.^32^ Genetic data for atypical Parkinsonism disorders remain sparse and are largely limited to European populations. Whereas more than 150 common PD risk loci have been identified,^20^ fewer than 10 genes have been implicated in each atypical disorder,^33–35^ although monogenic cases have been reported for PSP, MSA, and CBD.^36^ The genetic architecture of atypical Parkinsonism, therefore, remains insufficiently characterized across both Europeans and URP.

Over the past year, GP2 advanced this effort by engaging multiple centres caring for patients with MSA, PSP, DLB, and CBS. The GP2-MSA initiative launched in June 2024 as an international collaboration across GP2 sites in Nigeria, the United Kingdom, and Malaysia, with at least 2000 genotyped MSA samples expected by 2026. In LATAM, the PANMSA study is also due to start MSA sample collection in 2026. Additional atypical Parkinsonism initiatives are planned, including a dedicated PSP program. Over the next five years, GP2 aims to collect 10,000 samples from individuals with atypical Parkinsonism for genotyping through existing and newly established global GP2 cohorts.

At the time of clinical PD diagnosis, approximately 75% of dopaminergic neurons in the substantia nigra have already been lost,^37^ indicating an advanced disease stage. For this reason, prodromal PD has become a major area of expansion for GP2. A large multicenter study demonstrated that more than 70% of individuals with polysomnography-confirmed isolated REM sleep behavior disorder (iRBD) developed parkinsonism within 12 years of follow-up.,^38^ most commonly converting to idiopathic PD, but also to DLB and MSA. Additional features such as hyposmia and subtle motor changes further increase conversion risk.^38^ Current risk algorithms, including PREDICT-PD, now incorporate clinically probable RBD and hyposmia as predictors of Parkinsonism.^39^ Genetic studies in iRBD have implicated variants in lysosomal pathway genes, including *GBA1*, *TMEM175*, and *SCARB2*, with additional associations at *SNCA* involving variants distinct from those typically associated with PD,^40,41^ although effect direction remains debated, and most data are from European populations. A recent study suggested roles for *SNCA* and *MAPT* in both hyposmia and PD,^42^ though reliance on self-reported olfactory status prevented assessment of bidirectional effects. More comprehensive studies are required, particularly in URP, to define the genetic landscape of prodromal Parkinsonism.

The prodromal phase is increasingly central to clinical research design. GP2's prodromal effort therefore prioritizes (1) well-characterised clinical cohorts, including individuals with RBD, hyposmia, and pure autonomic failure (PAF), and (2) genetic at-risk cohorts, such as non-manifesting carriers of *GBA1* and *LRRK2* variants, as well as biologically defined cohorts identified through α-synuclein seed amplification assays (SAA). Over the next year, GP2 aims to generate genotyping data for 10,000 prodromal participants globally, along with harmonized clinical data, and to establish new cohorts in Africa, Asia, and Latin America. Within four years, the goal is to expand to 20,000 genotyped prodromal participants worldwide. Current prodromal research criteria combine clinical phenotypes with genetic and biological markers, aligned with frameworks such as SyNeurGe and NSD-ISS.^43,44^ Genetic stratification is increasingly integral to clinical trial design, highlighted by ongoing international trials focusing on carriers of *GBA1* and *LRRK2* variants.^45,46^

### Integrating complex and monogenic genetic assessments

Genotyping has traditionally been used to investigate the complex genetic architecture of PD by evaluating common variants associated with disease risk. However, several PD-associated genes harbor both rare variants that cause familial PD and common variants that influence sporadic PD risk. For example, *LRRK2* variants account for up to 4–36% of familial PD in certain populations and contribute to approximately 1–39% of sporadic PD, depending on ancestry.^47^ Similarly, *SNCA*, the first PD gene discovered, has a dual role, with rare variants causing familial PD,^48^ and common variants modulating risk for sporadic PD.^49^ These examples illustrate the concept of pleomorphic risk loci, in which a single gene harbors both rare pathogenic variants and common risk variants, reinforcing the idea of a biological continuum between monogenic and complex PD.^50^ The NeuroBooster Array (NBA) is a high-density genotyping platform designed to capture both common and rare genetic variation at scale.^51^ NBA-based analyses rely on imputation, which currently performs best for individuals of European ancestry; however, ongoing efforts are expanding reference panels and improving imputation performance across diverse global populations.^52^

As sequencing technologies become more accessible and cost-effective, GP2 is expanding beyond NBA-based genotyping to include WGS at scale. Initially intended primarily for monogenic cases, WGS is now being applied more broadly, with 38,226 genomes sequenced to date and a target of 100,000 individuals. This strategy will enhance genomic coverage, particularly in underrepresented and admixed populations.

## Capacity building and training development

Another core mission of GP2 is to train the next generation of PD researchers and build global research capacity. The GP2 Training and Networking WG leads this effort by providing resources and expertise to meet the diverse training needs.

To support capacity building, GP2 has developed a suite of comprehensive, self-directed training materials designed to accommodate researchers and clinicians across diverse levels of expertise and backgrounds. These resources encompass a broad range of topics, including theoretical and practical aspects such as sample preparation, shipping protocols, clinical applications, research methodology, and both basic and advanced bioinformatics. Importantly, GP2 continually updates these curricula based on feedback from members, especially those from URPs, to address training gaps and meet local needs. Specific content areas include complex genetics (e.g., GWAS, polygenic risk scores, case-control single-gene analysis, array-based copy number variant pipelines, haplotype analysis, etc.) as well as WGS applications (e.g., *GBA1* variant screening via the ‘Gauchian’ pipeline, structural variation detection, repeat expansion analysis, etc.).

The training materials are available in various formats, including recorded lectures, interactive modules, demonstration videos, and executable notebooks, and are freely accessible to all users who register an account on the GP2 Learning Platform (training.gp2.org). To date, over 1500 individuals have registered and engaged with these resources, reflecting the broad and growing global participation in this platform. All analytical workflows are hosted on the Terra (https://app.terra.bio/) and Verily cloud-based platforms (https://verily.com/solutions/pre-platform/workbench), enabling scalable access to GP2 and AMP-PD datasets that can be readily analysed using integrated or built-in tools.^53^ In the long run, this secure member-access model supports equitable learning opportunities and encourages broad participation of GP2 collaborators in PD genetics research worldwide.

### Engagement and reach-out to trainees worldwide

Aligned with its vision to build a globally connected research community, the GP2 Trainee Network primarily supports early-career researchers, including clinicians, geneticists, and data scientists, by providing opportunities to gain and strengthen research skills within a collaborative international environment. The network has historically included approximately 410 members, with around 130 current active trainees from diverse geographic, academic, and professional backgrounds. To facilitate broad and inclusive participation, regional trainee groups from Africa, Latin America, Asia, Australasia, and Central and Eastern Europe have been established under this initiative, led by GP2 trainee representatives in each region. These regional groups serve as hubs for localized mentorship, peer support, and tailored training activities that address region-specific research priorities and resource needs. This decentralized mechanism empowers trainees to take leadership roles in their communities, strengthens local research networks, and, in the long run, amplifies the global impact of GP2's training efforts.

To support outreach and engagement among trainees, GP2 routinely organizes training workshops tailored to local research priorities and the specific needs of trainees in each region. These in-person regional workshops provide a comprehensive learning experience, offering hands-on training in both bioinformatics analyses and the clinical aspects of PD. GP2 also organizes hackathons that bring together trainers and trainees to apply their technical skills in collaborative, data-driven projects. During these events, participants work on GP2 datasets to address PD–related research questions, leading to publishable outputs and advancing computational methods and data analysis pipelines within the consortium.^54^ These hackathons offer a unique opportunity for early-career researchers to strengthen problem-solving and teamwork skills while contributing directly to GP2's global research goals.

**Figure 2.**
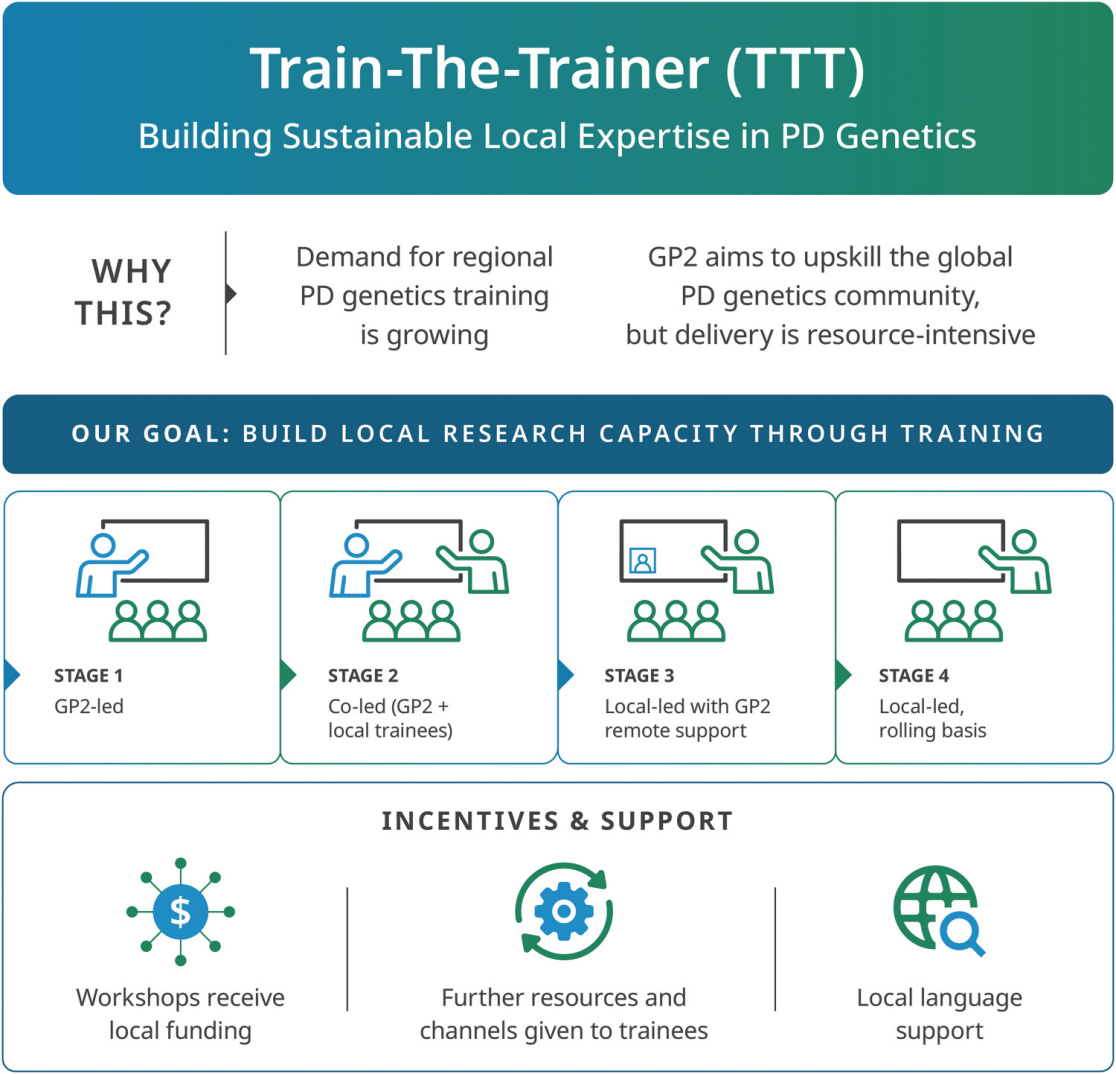
Train-The-Trainer (TTT) framework within GP2. The schematic illustrates a phased capacity-building strategy for Parkinson's disease (PD) genetics training. Workshops are initially delivered by GP2 trainers, followed by joint facilitation with local trainees. Trainees then progress to independently leading workshops with remote GP2 oversight, ultimately achieving full local autonomy in training delivery.

A “Train The Trainer” (TTT) approach is designed to foster mentorship and sustain knowledge dissemination within GP2 (Figure 2). This stepwise mechanism supports trainees’ transition into teaching roles by providing structured mentorship, teaching opportunities, and resources to deliver local and regional training or workshops. By empowering trainees to become local trainers and future leaders, GP2 seeks to cultivate a scalable and diverse global workforce, thereby enhancing global research capacity in PD genetics.

During the consortium's initial phase, trainee activities focused on building writing skills, resulting in 23 trainee-led publications, including five literature reviews, a book chapter, and nine “Hot Topic” articles.^55–59^ These publications spanned topics ranging from global diversity in PD genetics to molecular mechanisms such as DNA methylation in Lewy body pathology and mitochondrial dysfunction.^60–62^ As GP2's multi-ancestry datasets became available, trainee-led efforts shifted toward data-driven projects, yielding two research articles examining PD PRS across ancestries^63^ and the shared genetic architecture of PD and depression, among other works.^64^ Looking ahead, 29 ongoing projects span single-gene assessments, analyses of PD loci across diverse ancestries, and studies of comorbidity and prodromal features.^65–68^

Executable notebooks developed as part of training programs or projects are continuously and systematically archived in Zenodo (https://zenodo.org/) and GitHub (https://github.com/GP2code). This infrastructure not only facilitates seamless integration of training and data analysis but also ensures transparency, reproducibility, and long-term accessibility of training outputs. Collectively, this growing network of GP2 trainees highlights how strategic investment in training and mentorship not only builds global research capacity but also drives discovery across the full spectrum of PD genetics.

### Support for postgraduate programs, courses and conferences

**Figure 3.**
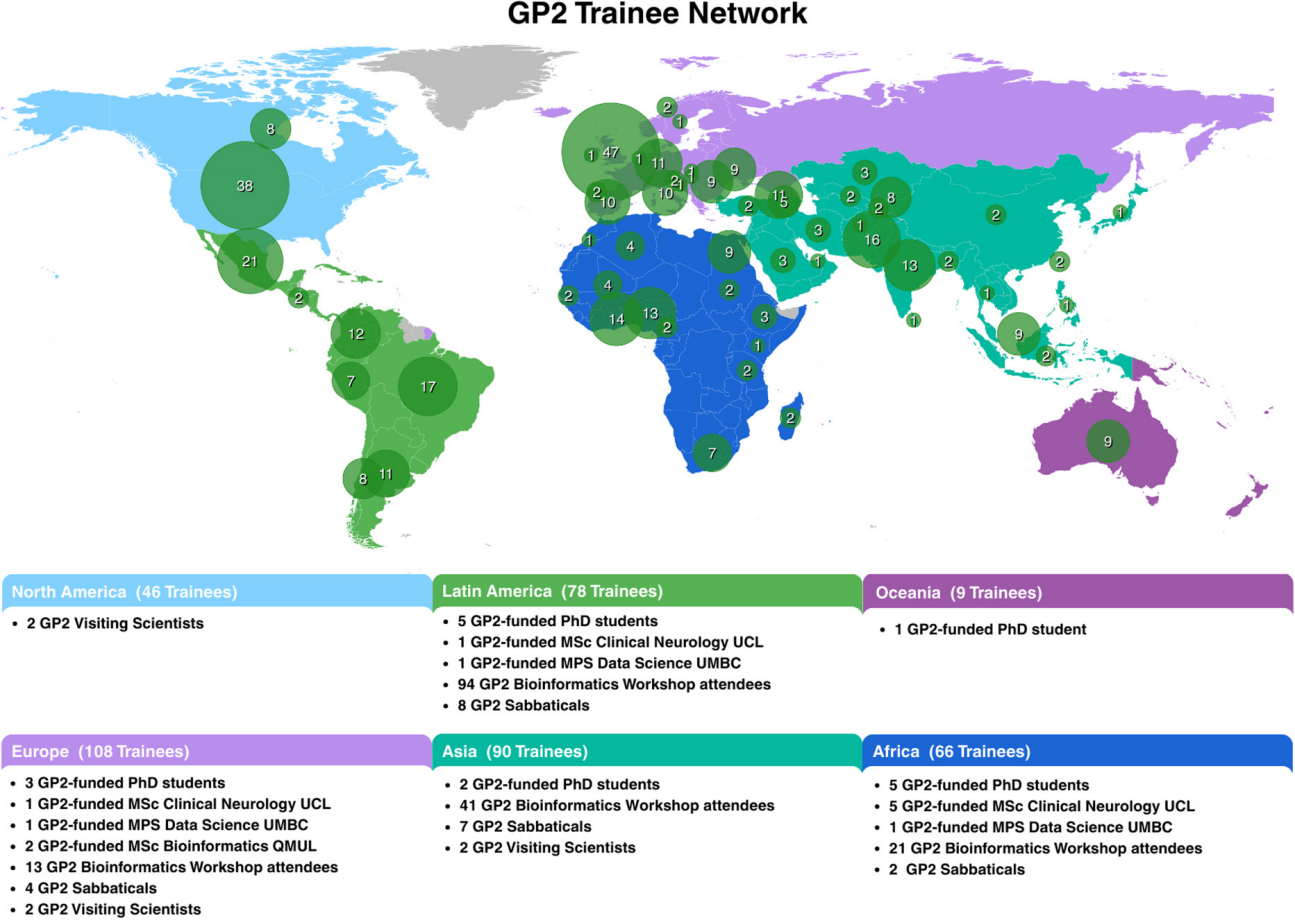
Global distribution of GP2 trainee participation and funded training opportunities. The world map shows the geographic distribution of GP2 trainees by country. Bubble size is proportional to the number of trainees per country, with numeric labels indicating trainee counts. Countries are coloured by broad geographic region. Summary boxes report the total number of trainees per region and the corresponding GP2-facilitated training opportunities, including funded PhD and MSc positions, visiting scientist placements, bioinformatics workshop participation, and sabbatical support.

In addition to training workshops, GP2 provides financial support for postgraduate education, including funding for PhD and MSc students, particularly from URP. To date, GP2 has supported 19 PhD and 11 MSc students from Africa, Asia, Australia, and Latin America (Figure 3), with projects covering a wide range of topics in PD and aiming to advance our understanding of PD genetics in URP. GP2 also allocates funding for research site visits and sabbatical programs on a rolling basis, enabling trainees and researchers to gain hands-on experience in partner institutions within GP2. These opportunities are designed to promote knowledge exchange, build collaborative networks, and equip trainees with the skillsets needed in conducting analyses centred on PD research. Applications for these programs have been offered annually during the GP2 initiative, though current PhD and MSc calls are now closed.

GP2 stands as a transformative, collaborative initiative that has redefined the landscape of PD genetics through equitable data sharing, capacity building, and global engagement. By integrating monogenic and complex genetic analyses across ancestrally diverse populations, GP2 has expanded the understanding of PD's genetic architecture while fostering a new generation of researchers equipped to lead future discoveries. As the program grows and evolves, its growing global network, open-science infrastructure, and commitment to inclusivity will continue to accelerate translational insights, shaping the path toward precision medicine and a more comprehensive understanding of the Parkinsonian spectrum worldwide.
